# Agreement Between Automated Pedicle Screw Planning and Surgeon-Selected Implant Dimensions in Thoracic and Lumbar Spine Surgery: A Three-Year Single-Center Study

**DOI:** 10.3390/jcm15155804

**Published:** 2026-07-24

**Authors:** Laura Herkner, Franz-Josef Hans, Mihail-Lucian Stefan, Joachim K. Krauss, Shadi Al-Afif, Sami Ridwan

**Affiliations:** 1 Department of Neurosurgery, Hannover Medical School, Carl-Neuberg-Straße 1, 30625 Hannover, Germany; 2 Department of Neurosurgery, Klinikum Ibbenbueren, Große Str. 41, 49477 Ibbenbueren, Germany

**Keywords:** automated planning, pedicle screw, spine navigation, implant dimensions, Bland–Altman analysis

## Abstract

**Background:** Automated pedicle screw planning (ASP) can initialize trajectories and implant dimensions, but correlation with implanted screws does not establish agreement. We quantified the association and agreement between ASP recommendations and actual intraoperative implant selection (AIS) and examined pathology, asymmetric planning, and available safety outcomes. **Methods:** This retrospective single-center study included 103 consecutive patients treated with thoracic or lumbar instrumentation from January 2021 to January 2024. The dataset contained 351 bilateral screw-pair records. Pearson and Spearman correlations, linear regression, and Bland–Altman analyses were supplemented by patient-clustered bootstrap 95% confidence intervals (CIs), pathology-stratified estimates, and a sensitivity analysis excluding a verified 94 mm iliac-fixation observation. **Results:** Diameter showed moderate correlation (Pearson’s r = 0.635, 95% CI 0.542–0.721; Spearman’s rho = 0.679, 95% CI 0.554–0.767; both *p* < 0.001). Length also showed moderate correlation (Pearson’s r = 0.675, 95% CI 0.565–0.754; Spearman’s rho = 0.600, 95% CI 0.450–0.714; both *p* < 0.001). For ASP minus AIS, the diameter bias was −0.75 mm (95% CI −0.86 to −0.64), with limits of agreement from −2.00 to 0.50 mm. The length bias was −0.15 mm (95% CI −1.05 to 0.79), with limits from −12.30 to 12.00 mm. The 94 mm value was verified as a genuine iliac-fixation observation; excluding it changed Pearson’s r only from 0.675 to 0.679. Asymmetric ASP length recommendations occurred in 115/351 pairs (32.8%), usually by 5 mm. **Conclusions:** ASP tracked overall implant-size trends but did not provide interchangeable estimates of surgeon-selected dimensions. Its systematic diameter underestimation and broad pair-level differences support its use as an initialization and review aid, with final sizing retained as a surgeon decision.

## 1. Introduction

Pedicle screw fixation is used across cases of degenerative disease, trauma, infection, and neoplasia. Safe fixation requires a trajectory that remains within the osseous corridor and an implant whose diameter and length provide adequate purchase without unacceptable cortical, neural, or vascular risk. Navigation improves placement accuracy in many settings, yet implant size remains clinically important because both the insertion depth and diameter affect the bone–screw interface and mechanical stability [1,2,3,4,5,6,7,8].

Contemporary navigation platforms increasingly include automated screw planning (ASP). After CT import, these tools identify vertebral levels, segment or register the bony anatomy, construct candidate transpedicular trajectories, and propose a screw diameter and length. Automated initialization may reduce repetitive planning work and provide a consistent starting point, especially in multilevel procedures. Nevertheless, the output remains dependent on the imaging data, anatomical model, safety constraints, trajectory rules, and available implant increments [9,10,11,12,13].

Most clinical navigation studies have evaluated cortical breach or placement accuracy rather than whether an automated size recommendation matches the implant ultimately selected by the surgeon. The distinction matters. A trajectory can be geometrically acceptable even when its proposed diameter or length differs from the surgeon’s choice, and a system that produces highly correlated values can still show clinically relevant systematic bias or wide limits of agreement. Recent automated-planning studies have compared proposed trajectories with expert plans or freehand implants, but evidence from consecutive routine cases and heterogeneous pathologies remains limited [10,11,12,13,14].

Correlation and agreement therefore answer different questions. Correlation indicates whether larger automated recommendations tend to accompany larger implanted screws; it does not show that the two methods are interchangeable. Bland–Altman analysis directly quantifies the mean paired difference and the range containing most individual differences. Reporting both approaches is particularly important when software is intended to support decisions at the level of an individual vertebra rather than to only describe a cohort-level trend.

Clinical practice adds information that is not fully represented by a preoperative CT-based plan. Surgeons may respond to the cortical integrity, bone quality, tactile feedback during pedicle preparation, pathology-related destruction, augmentation strategy, selected trajectory, and size increments of the implant system. Infection, tumor, and trauma can also distort the anatomy or degrade segmentation and registration. These considerations provide plausible mechanisms for disagreement and justify pathology-stratified and sensitivity analyses.

The primary objective of this study was to quantify the correlation and agreement between ASP-derived screw diameter and length and actual intraoperative implant selection (AIS) in consecutive thoracic and lumbar instrumentation cases. Secondary objectives were to examine whether agreement varied across pathology groups, characterize left–right asymmetry in automated length planning, evaluate the influence of a verified 94 mm iliac-fixation observation, and descriptively relate the available postoperative safety observations to ASP-AIS discrepancies.

## 2. Materials and Methods

### 2.1. Study Design and Patient Population

This retrospective observational study was conducted at a single neurosurgical center and included consecutive thoracic and lumbar instrumentation procedures performed between January 2021 and January 2024 for which ASP was available. A total of 103 patients met the inclusion criteria. Rather than a predominantly degenerative sample, the cohort represented a heterogeneous and comparatively complex case mix: spinal infection was the largest individual category (29.1%), while degenerative disease and trauma each accounted for 24.3% and tumor for 22.3%.

Demographic and clinical variables were obtained from institutional records and included age, sex, urgency, number of instrumented segments, and underlying pathology. Procedural characteristics included percutaneous or open implantation, polymethylmethacrylate (PMMA) augmentation, decompression, fusion, and the use of carbon fiber-reinforced polyetheretherketone (carbon–PEEK) implants. The available perioperative variables included postoperative CT, pedicle violation, screw revision, durotomy, and wound revision.

### 2.2. Automated Screw Planning Workflow

Automated planning was performed with Brainlab Elements SpinePlanning 2.0 (Brainlab SE, Munich, Germany), which is built on an atlas-based algorithm. Preoperative thin-slice CT data (slice thickness 0.5–1.0 mm) were imported, and the visible spine was automatically segmented. Vertebral-level labels were reviewed and approved or corrected. The system then generated bilateral trajectories and proposed a screw diameter and length. These unmodified automated values were archived before any surgeon adjustment, thereby preserving the independent ASP recommendation. A PDF report linked each proposed size to the vertebral level and side. Magnetic resonance imaging and dynamic radiographs were available when clinically indicated, but the automated dimensions were generated from CT data.

### 2.3. Surgeon Review and Intraoperative Implant Selection

The purely software-generated ASP plan was saved before surgeon review. The operating surgeon then modified the plan as considered appropriate and saved the revised plan separately. Screw dimensions were therefore changed during preoperative planning rather than in response to intraoperative tactile feedback. This distinction was particularly relevant for the many minimally invasive procedures, in which tactile assessment was not a standardized basis for changing screw dimensions. Navigated instruments were used in some, but not all, procedures. All planning and procedures were performed by one of two specialist spine surgeons. Final implanted dimensions were verified from the mandatory implant documentation in the electronic hospital database, including the implant system and recorded screw dimensions.

### 2.4. Data Extraction and Outcome Definitions

Each analytical record represented a bilateral pedicle screw pair at one planned vertebral level. The final dataset comprised 351 pair-level records from 103 patients, corresponding to approximately 700 individual screws. ASP and AIS dimensions were recorded in millimeters. Paired differences were defined throughout as ASP minus AIS; negative values therefore indicate that ASP recommended a smaller dimension than the implanted screw. Pathology was grouped as degenerative disease, infection, trauma, or tumor.

Automated length asymmetry was defined as different left and right ASP length recommendations at the same vertebral level. The absolute left–right difference was calculated for asymmetric plans. When ASP proposed unequal bilateral lengths, the longer of the two automated screw lengths was retained as the principal ASP value for comparison with the pair-level AIS length recorded in the source dataset.

Postoperative CT status and complications were summarized at the patient level. Because only one patient had a documented pedicle violation and no patient underwent screw revision, clinical outcomes were not subjected to inferential modeling. Instead, absolute ASP-AIS differences in the violation case were compared descriptively with the remaining records. Durotomy and wound revision were treated as broader perioperative outcomes rather than events attributable to screw-size disagreement.

### 2.5. Statistical Analysis

Continuous variables are presented as the mean and standard deviation or median and range, as appropriate; categorical variables are reported as the number and percentage. Pearson correlation quantified linear association, Spearman rank correlation assessed monotonic association and robustness to non-normality and influential values, and simple linear regression described the relationship between ASP and AIS. Two-sided *p* values below 0.05 were considered statistically significant.

Agreement was evaluated using Bland–Altman analysis. For each dimension, the mean paired difference (bias), standard deviation of the differences, and lower and upper limits of agreement (bias ± 1.96 standard deviations) were calculated. The sign convention was ASP minus AIS. Correlation coefficients, regression slopes, bias, and limits of agreement are reported with 95% confidence intervals.

Because several pair-level measurements arose from the same patient, confidence intervals were estimated with a patient-clustered percentile bootstrap. Patients, rather than individual screw-pair records, were sampled with replacement; all available records from a selected patient were retained in each bootstrap sample. Ten thousand replicates were used with a fixed random seed. This approach preserved within-patient dependence and avoided treating all 351 records as independent for interval estimation.

Prespecified secondary analyses calculated pathology-specific bias and limits of agreement for degenerative disease, infection, trauma, and tumor. Given the modest number of patients within each subgroup, these analyses were interpreted descriptively. A sensitivity analysis repeated the length correlation, regression, and Bland–Altman calculations after excluding the single record with ASP length 94 mm and AIS length 60 mm. Source review confirmed that the 94 mm value was a genuine iliac-fixation observation rather than a data-entry, export, or matching error. It was retained in the primary analysis, while the sensitivity analysis assessed its influence because iliac fixation differs anatomically from standard thoracolumbar pedicle fixation.

Original descriptive and correlation outputs were generated in IBM SPSS Statistics version 25.

## 3. Results

The analysis included 103 patients with a mean age of 67.6 ± 13.8 years (median 69, range 27–89); 54 were female and 49 male. The case mix was relatively balanced across infection, degenerative disease, trauma, and tumor, with infection being the largest category. Of 351 screw-pair records, 250 (71.2%) were implanted percutaneously, 70 (19.9%) were cement-augmented, and 73 (20.8%) used carbon–PEEK implants. The implant systems were VADER (icotec ag, Altstaetten, Switzerland), RELINE (Globus Medical, Bremen, Germany), and Ennovate (B. Braun SE, Melsungen, Germany). Characteristics are summarized in Table 1.

The 351 bilateral pair-level records comprised 59 records from degenerative cases, 98 from infection, 97 from trauma, and 97 from tumor cases. Thoracic and lumbar levels were represented, providing a range of pedicle and vertebral-body dimensions. The analysis did not treat the approximately 700 individual screws as independent observations.

The mean ASP diameter was 5.24 ± 0.60 mm (median 5.50; range 3.50–7.50), compared with 5.99 ± 0.82 mm for AIS (median 6.50; range 4.50–8.50). The mean ASP length was 46.90 ± 8.27 mm (median 45; range 25–94), compared with 47.05 ± 6.66 mm for AIS (median 50; range 25–70).

**Figure 1:**
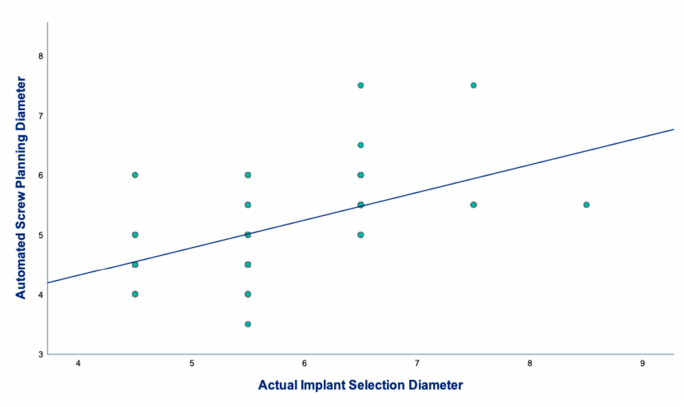
A scatter plot of ASP and AIS screw diameters. A moderate association (Pearson’s r = 0.635, 95% CI 0.542–0.721; *p* < 0.001) coexists with systematic ASP underestimation shown by the agreement analysis.

Diameter showed a moderate positive association between ASP and AIS. Pearson’s r was 0.635 (95% CI 0.542–0.721), and Spearman’s rho was 0.679 (95% CI 0.554–0.767); both *p* values were <0.001 (Figure 1 and Table 2). Thus, larger automated diameters generally accompanied larger implanted diameters, but the correlation did not establish agreement for an individual pair.

**Figure 2:**
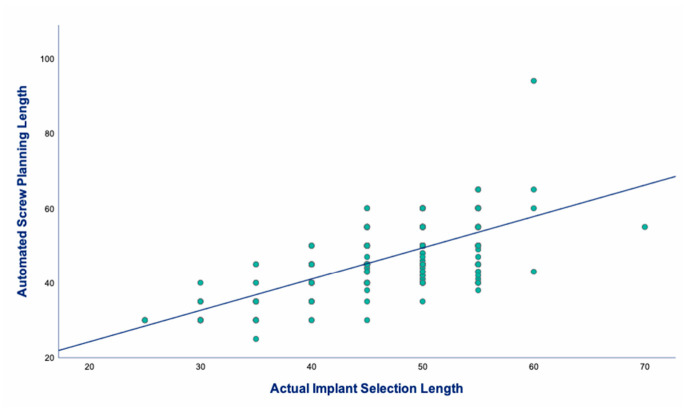
A scatter plot of ASP and AIS screw lengths, including the verified 94 mm iliac-fixation observation. Pearson’s r = 0.675 (95% CI 0.565–0.754) and Spearman’s rho = 0.600 (95% CI 0.450–0.714); both *p* < 0.001.

Length also showed a moderate association: Pearson’s r was 0.675 (95% CI 0.565–0.754), and Spearman’s rho was 0.600 (95% CI 0.450–0.714); both *p* values were <0.001 (Figure 2 and Table 2). Source review confirmed that the 94 mm ASP value represented genuine planning for iliac fixation and was not a data-entry, export, or matching error. The observation was retained in the primary analysis. After its exclusion, Pearson’s r was 0.679, Spearman’s rho was 0.596, and the regression slope changed from 0.543 to 0.571, indicating no material influence on the overall conclusion.

Linear regression remained significant for both dimensions (*p* < 0.001). For diameter, AIS = 1.439 + 0.868 × ASP (slope 95% CI 0.684–1.086; R2 = 0.403). For length, AIS = 21.575 + 0.543 × ASP (slope 95% CI 0.423–0.654; R2 = 0.455). The residual dispersion in both models reinforces that regression prediction and method agreement are not equivalent.

**Figure 3:**
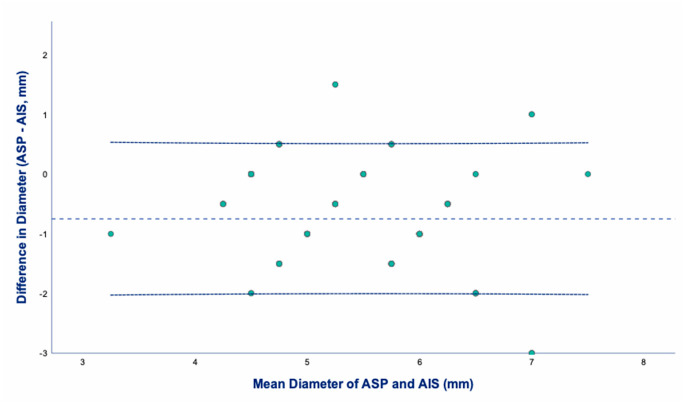
Bland–Altman plot for screw diameter. Differences are ASP minus AIS. Bias was −0.75 mm and 95% limits of agreement were −2.00 to 0.50 mm.

**Figure 4:**
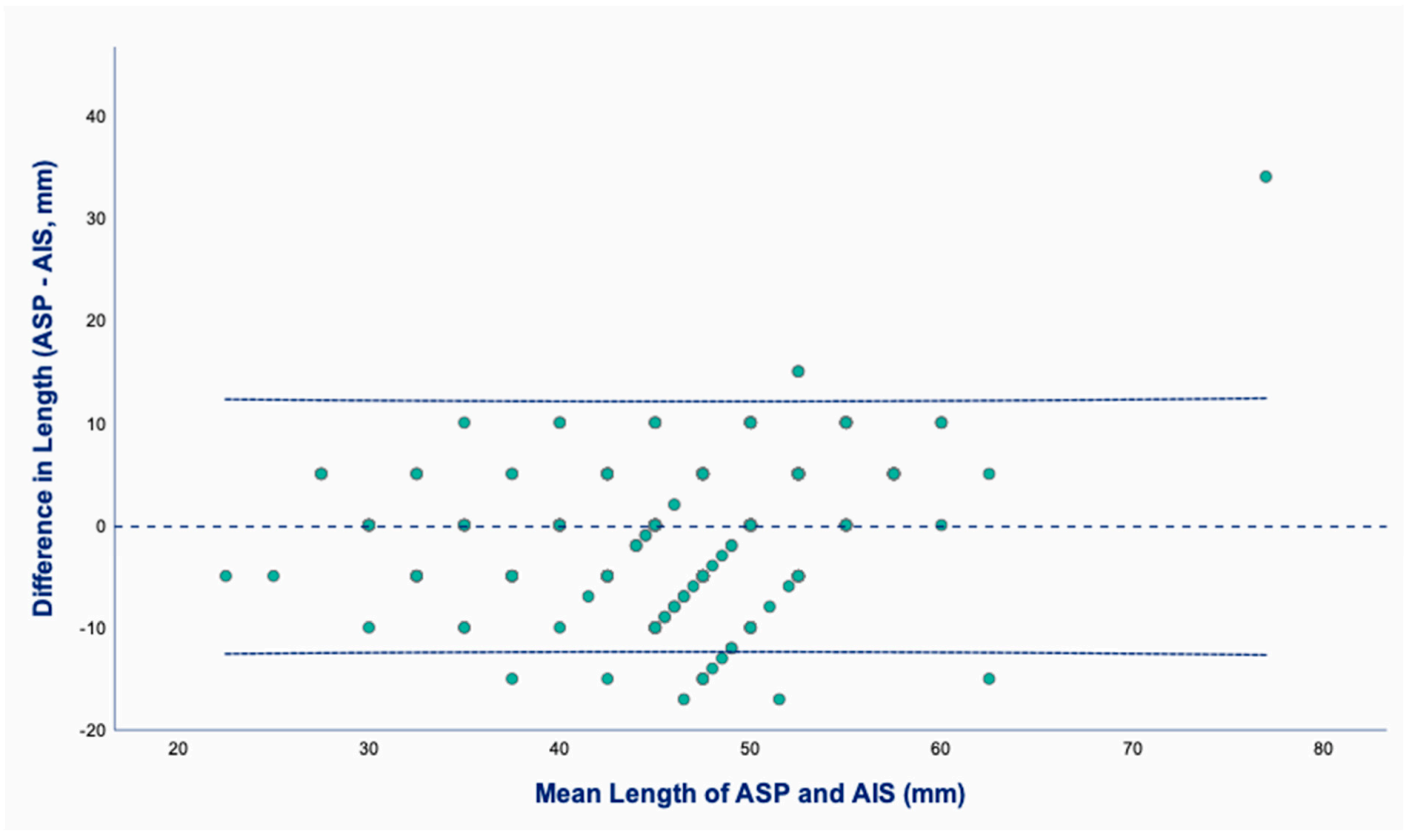
Bland–Altman plot for screw length. Differences are ASP minus AIS. Bias was −0.15 mm and 95% limits of agreement were −12.30 to 12.00 mm; +34 mm difference corresponds to verified 94 mm iliac-fixation observation.

**Figure 5:**
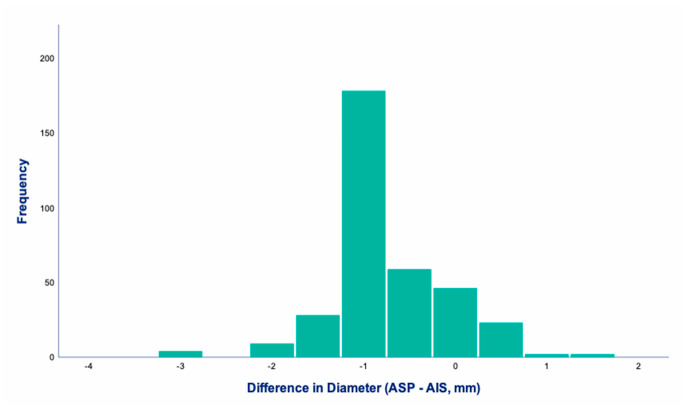
The distribution of diameter differences (ASP minus AIS). The negative shift is consistent with a mean bias of −0.75 mm.

Bland–Altman analysis quantified the disagreement directly (Figure 3 and Figure 4; Table 3). The diameter bias was −0.75 mm (95% CI −0.86 to −0.64; Figure 5), with a difference standard deviation of 0.64 mm and limits of agreement from −2.00 mm (95% CI −2.22 to −1.80) to 0.50 mm (95% CI 0.25–0.72). ASP therefore recommended a smaller diameter on average, while individual pair differences extended from approximately 2 mm smaller to 0.5 mm larger than AIS.

Length showed little average directional bias but considerable pair-level dispersion. The bias was −0.15 mm (95% CI −1.05 to 0.79), the difference standard deviation was 6.20 mm, and the limits of agreement were −12.30 mm (95% CI −14.11 to −10.47) and 12.00 mm (95% CI 10.17–14.07). Excluding the verified 94 mm iliac-fixation observation shifted the bias to −0.25 mm and narrowed the limits modestly to −11.88 and 11.38 mm. The small mean bias should therefore not be interpreted as close agreement for every pair.

ASP proposed unequal left and right lengths in 115/351 bilateral pairs (32.8%). The median absolute asymmetry was 5 mm (IQR 5-5; range 5–10). Asymmetry occurred in 18/59 degenerative pairs (30.5%), 37/98 infection pairs (37.8%), 31/97 trauma pairs (32.0%), and 29/97 tumor pairs (29.9%) as shown in Table 4. The verified 94 mm iliac-fixation observation was not an asymmetric plan. No unequal left–right ASP diameter values were recorded. These results are presented descriptively because the source dataset did not contain side-specific AIS dimensions suitable for a direct left–right agreement analysis.

No screw revision was required. Postoperative CT was available for 102/103 patients and documented one pedicle violation. That patient contributed two pair-level records: the absolute diameter difference was 1.0 mm in both, and the absolute length difference was 0 mm in both. For all remaining records, the median absolute differences were 1.0 mm for diameter and 5.0 mm for length. The event count precluded inferential modeling and did not suggest that the single violation occurred in a case with unusually large recorded disagreement. Durotomy occurred in 12 patients (11.7%) and wound revision in six (5.8%); these were retained as general perioperative outcomes and were not attributed to ASP-AIS size differences.

## 4. Discussion

### 4.1. Principal Findings

In this consecutive, heterogeneous cohort, automated and implanted dimensions moved together at a group level, but neither diameter nor length showed agreement sufficient to make ASP and AIS interchangeable. The central quantitative finding was a −0.75 mm diameter bias, with 95% limits from −2.00 to 0.50 mm. Length had almost no average bias, yet individual differences commonly spanned a clinically relevant range, with limits of approximately −12 to 12 mm. Patient-clustered confidence intervals confirmed the precision of these estimates without an assumption of independence among multiple records from the same patient.

### 4.2. Potential Explanations for Diameter Underestimation

The direction of diameter disagreement is consistent with a conservative automated planning rule. Published planning methods commonly derive the diameter from the minimum distance between a candidate centerline and the pedicle cortex, then round down to an available size to preserve a safety margin [9,10]. A plan based on the inner cortical corridor may therefore be smaller than a screw selected by a surgeon who accepts controlled cortical engagement, modifies the trajectory, or prioritizes purchase in weak bone. Diameter and insertion depth influence fixation strength, so a larger implant can be a deliberate intraoperative choice rather than evidence that the software generated an anatomically unsafe plan [7,8].

Several additional mechanisms may contribute. CT voxel size and partial-volume effects can shift an estimated cortical boundary; registration or segmentation can be less stable in cases of deformity, previous surgery, fracture, infection, or tumor; and even a small trajectory change can alter the narrowest pedicle corridor [9,10,11,12]. Because the software is atlas-based, correspondence between patient anatomy and the registered atlas may also contribute. The bone quality, augmentation strategy, and discrete implant sizes may further influence surgeon selection. These mechanisms remain plausible rather than proven because the software’s proprietary internal sizing and trajectory rules are unavailable. The present results therefore support systematic underestimation.

The direction of disagreement is not necessarily universal across automated systems. In one clinical evaluation, AI-selected screws were larger and longer than freehand implants, whereas registration-based comparisons have shown that surgeons often retain or adjust automated proposals by one catalog increment [13,14]. Algorithm design, reference trajectories, implant libraries, and local surgical practice can therefore determine whether an automated recommendation appears conservative or aggressive. External validation should report dimensional agreement for each software version and clinical setting rather than extrapolate from placement accuracy alone.

### 4.3. Length Agreement and the Iliac-Fixation Observation

Length illustrates why a small mean bias should not be equated with individual agreement. The ASP and AIS means were almost identical, but the standard deviation of paired differences was 6.20 mm and the limits approached ± 12 mm. A 5 mm difference corresponds to a common catalog increment and was the median absolute discrepancy. The rank correlation for length (rho = 0.600) was lower than Pearson’s r, consistent with the discrete and non-normal distribution of sizes, but remained moderate and statistically significant. The 94 mm value was confirmed as genuine planning for iliac fixation, where the anatomical target and expected screw-length range differ from those for standard thoracolumbar pedicle fixation. Excluding it changed the correlation, regression, bias, and limits only modestly; therefore, it did not drive the overall conclusion.

### 4.4. Pathology and Asymmetric Planning

The pathology analysis did not confirm the proposed explanation that infection and tumor necessarily produce the greatest diameter disagreement. Mean diameter underestimation was largest in degenerative cases (−1.12 mm), followed by infection (−0.82 mm), tumor (−0.68 mm), and trauma (−0.53 mm). Length bias varied in its direction, while all pathology groups retained broad limits of agreement. These subgroup estimates are descriptive: each category contained only 23–30 patients; pathology was confounded with the level, technique, augmentation, and implant system; and multiple records arose from each patient. The results are more useful for generating hypotheses than for claiming a pathology-specific software effect.

Asymmetric automated length planning deserves separate attention. Nearly one third of bilateral pairs had different left and right recommendations, usually by a single 5 mm increment, and the frequency was similar across pathology groups. Genuine pedicle or vertebral-body asymmetry, rotation, unilateral destruction, or independent trajectory optimization could explain this pattern. Alternatively, side-specific segmentation, rounding near a catalog threshold, or different endpoints along independently generated centerlines could produce unequal lengths. The absence of diameter asymmetry may reflect a different rounding or constraint rule for diameter rather than anatomical symmetry. Software documentation and side-specific AIS data are required to distinguish these mechanisms.

### 4.5. Clinical Relevance and Available Outcomes

The available safety data do not establish a clinical outcome effect. No screw was revised, and the single postoperative pedicle violation occurred in a patient whose recorded pair-level discrepancies were not larger than those for the rest of the cohort. With one event, neither equivalence nor risk association can be inferred. Durotomy and wound revision are broader procedural outcomes with many causes and should not be presented as consequences of screw-size disagreement. Accordingly, the revised title and conclusions emphasize correlation and agreement rather than claiming clinical reliability from sparse complication data.

### 4.6. Practical Applications and Future Development

In practice, ASP can still provide value as an initialization and quality-control tool. A proposed plan can standardize the first review of a multilevel case, expose unusual dimensions for deliberate confirmation, and provide a documented baseline against which surgeon modifications are assessed. The present data suggest two useful safeguards: an automated diameter should not be accepted without evaluating whether its conservative bias is appropriate for the planned trajectory and bone quality, and large length discrepancies or left–right asymmetry should trigger image review rather than automatic acceptance.

Future systems could combine the osseous corridor with quantitative CT surrogates of bone quality, pathology labels, implant-system constraints, and the history of accepted surgeon modifications. Prospective development should separate algorithm errors from reasonable surgeon preferences by recording the initial automated proposal, every manual change in trajectory and size, the intraoperative reason for the change, and side-specific implanted dimensions. Multicenter validation should report agreement and calibration by vertebral region, pathology, software version, and implant catalog, followed by clinically meaningful outcomes such as breach, loosening, revision, and construct failure.

### 4.7. Limitations

This study is retrospective and reflects one center, two experienced spine surgeons, three implant systems, and one commercial planning platform. The cohort’s heterogeneous pathology improved clinical broadness but introduced confounding, and subgroup sizes limited precise comparisons. The analytical unit was a bilateral pair-level record rather than independently documented left and right screws, which constrained interpretation of asymmetric planning and prevented a side-specific outcome analysis. When ASP lengths differed bilaterally, the longer automated value was used for the principal comparison; this pair-level rule does not substitute for a side-specific agreement analysis.

Automated planning was performed with an atlas-based platform; its proprietary internal sizing and trajectory rules were unavailable for this study. Screw dimensions were modified in the separately saved surgeon-reviewed preoperative plan, but navigated instruments were not used in every procedure, and intraoperative tactile feedback was not a standardized sizing method in the many minimally invasive cases. The analysis also included one verified iliac-fixation observation, although sensitivity analysis showed that it did not materially affect the length results. Although the clustered bootstrap statistically addressed repeated records, Bland–Altman limits still described the observed pair-level data structure rather than individual side-specific screws. Postoperative CT was nearly complete, but clinical events were too sparse for modeling, and longer-term outcomes such as loosening or fusion were not available. The exploratory subgroup analyses were not adjusted for the vertebral level, augmentation, technique, implant system, surgeon, or bone density.

Despite these limitations, this study addresses a practical question that the placement-accuracy literature does not answer: how closely an automated dimensional proposal resembles what surgeons implant. Consecutive inclusion, explicit agreement analysis, patient-clustered confidence intervals, sensitivity analysis, and pathology-specific estimates provide a reproducible quantitative basis for evaluating ASP as an adjunct rather than a substitute for clinical judgment.

## 5. Conclusions

Automated pedicle screw planning showed moderate correlations with surgeon-selected diameter and length but did not provide interchangeable pair-level estimates. ASP underestimated diameters by an average of 0.75 mm, with limits of agreement from −2.00 to 0.50 mm. The mean length bias was small, but limits of approximately −12 to 12 mm demonstrated substantial individual variation. The verified 94 mm iliac-fixation observation did not materially alter the findings, and asymmetric automated length planning occurred in 32.8% of pairs. ASP is best used to initialize and review a plan; the final trajectory and implant dimensions should remain subject to surgeon assessment and intraoperative findings.

## Figures and Tables

**Table 1:** Patient and surgical characteristics.

Clinical Data	
Number of patients	103
Median age (years)	69 (27–89)
Gender (female/male)	54/49
Number of screw pairs analyzed	351
Number of percutaneous screw pairs	250 (71.2%)
Number of cement-augmented screw pairs	70 (19.9%)
Number of carbon–PEEK screw pairs	73 (20.8%)
Median number of segments	3 (1–10)
Degenerative disease	25 (24.3%)
Spinal infection	30 (29.1%)
Tumor	23 (22.3%)
Trauma	25 (24.3%)
Elective surgery	92 (89.3%)
Decompression	87 (84.4%)
Fusion	51 (49.5%)

**Table 2:** Correlation between automated screw planning and actual implant selection. Confidence intervals account for clustering of records within patients.

Parameter	Pearson’s r (95% CI)	Spearman’s rho (95% CI)	*p*-Value
Screw diameter (n = 351)	0.635 (0.542–0.721)	0.679 (0.554–0.767)	<0.001
Screw length (n = 351)	0.675 (0.565–0.754)	0.600 (0.450–0.714)	<0.001

**Table 3:** Bland–Altman agreement estimates. Differences are defined as ASP minus AIS; confidence intervals account for patient-level clustering.

Parameter	Bias (95% CI), mm	Difference SD, mm	95% Limits of Agreement (95% CI), mm
Screw diameter	−0.75 (−0.86 to −0.64)	0.64	−2.00 (−2.22 to −1.80) to 0.50 (0.25 to 0.72)
Screw length	−0.15 (−1.05 to 0.79)	6.20	−12.30 (−14.11 to −10.47) to 12.00 (10.17 to 14.07)

**Table 4:** Pathology-specific agreement and asymmetric automated length planning. Bias is ASP minus AIS; subgroup confidence intervals use patient-clustered bootstrap resampling.

**Diameter**			
**Pathology**	**Patients/pairs**	**Bias (95% CI), mm**	**95% limits of agreement, mm**
Degenerative	25/59	−1.12 (−1.39 to −0.84)	−2.52 to 0.28
Infection	30/98	−0.82 (−0.98 to −0.64)	−1.91 to 0.28
Trauma	25/97	−0.53 (−0.72 to −0.34)	−1.70 to 0.64
Tumor	23/97	−0.68 (−0.91 to −0.45)	−1.87 to 0.50
**Length**			
**Pathology**	**Patients/pairs**	**Bias (95% CI), mm**	**95% limits of agreement, mm**
Degenerative	25/59	−1.78 (−3.34 to −0.12)	−12.33 to 8.77
Infection	30/98	0.88 (−0.50 to 2.29)	−12.46 to 14.21
Trauma	25/97	1.05 (−0.99 to 2.87)	−11.19 to 13.29
Tumor	23/97	−1.40 (−3.18 to 0.38)	−12.34 to 9.53

## Data Availability

De-identified data supporting the findings may be made available by the corresponding author upon reasonable request, subject to institutional approval and applicable privacy restrictions. Patient-level source data are not publicly available because they contain protected clinical information.

## Abbreviations

AIS	Actual intraoperative implant selection
ASP	Automated pedicle screw planning
CT	Computed tomography
PEEK	Polyetheretherketone
PMMA	Polymethylmethacrylate
